# Seed-to-seed early-season cold resiliency in sorghum

**DOI:** 10.1038/s41598-021-87450-1

**Published:** 2021-04-08

**Authors:** Yves Emendack, Jacobo Sanchez, Chad Hayes, Matthew Nesbitt, Haydee Laza, John Burke

**Affiliations:** 1 Cropping Systems Research Laboratory, USDA-ARS, 3810 4th Street, Lubbock, TX USA; 2 Department of Plant and Soil Sciences, Texas Tech University, Lubbock, TX USA

**Keywords:** Physiology, Plant sciences, Environmental sciences

## Abstract

Early planted sorghum usually experiences cooler day/night temperatures, which may result in delayed growth, floral initiation, and infertile pollen, limiting productivity in high altitudes and temperate regions. Genetic variability for cold tolerance in sorghum has been evaluated by characterizing germination, emergence, vigor, and seedling growth under sub-optimal temperatures. However, the compounded effect of early season cold on plant growth and development and subsequent variability in potential grain yield losses has not been evaluated. Agro-morphological and physiological responses of sorghum grown under early-, mid-, and standard planting dates in West Texas were characterized from seed-to-seed. A set of diverse lines and hybrids with two major sources of tolerance, and previously selected for seedling cold tolerance were used. These were evaluated with a standard commercial hybrid known for its seedling cold tolerance and some cold susceptible breeding lines as checks. Variabilities in assessed parameters at seedling, early vegetative, and maturity stages were observed across planting dates for genotypes and sources of cold tolerance. Panicle initiation was delayed, and panicle size reduced, resulting in decreased grain yields under early and mid-planting dates. Coupled with final germination percent, panicle width and area were significant unique predictors of yield under early and mid-planting dates. Significant variability in performance was observed not only between cold tolerant and susceptible checks, but noticeably between sources of cold tolerance, with the Ethiopian highland sources having lesser yield penalties than their Chinese counterparts. Thus, screening for cold tolerance should not be limited to early seedling characterization but should also consider agronomic traits that may affect yield penalties depending on the sources of tolerance.

## Introduction

Cold stress is a major factor in determining the natural distribution of plants^1^, phenology and yield potentials of agricultural crops^2^. One of the major limitations in expanding sorghum (*Sorghum bicolor* (L.) Moench) production into temperate and mountainous regions of the world is the potential to tolerate early-and-late season low temperatures. Cold tolerance, as defined by^3^, is the ability of crops to germinate, grow, and produce satisfactory yields under conditions of relatively cold (but above freezing) air and soil temperatures.

Though sorghum is noted for tolerance to hot and dry semi-arid environments, its gradual introduction into regions characterized by low temperatures has led to the evolution of cold tolerant lines^4–6^. Possible sources of cold tolerance have been linked to landraces that have evolved in the temperate regions of China^7^ and the highlands of Ethiopia, Rwanda and Uganda^8–10^. Studied genetic diversity in a collection of Chinese sorghum landraces have found that there exists significant diversity within these landraces for the seedling cold vigor trait. However, these Chinese landraces also harbor poor and undesirable agronomics with high grain tannin content which affects the feeding value^4,11^. Reported genetic diversity for seedling cold tolerance in converted Ethiopian lines. The Ethiopian sorghum germplasm collection is one of the largest collections in the world, with many lines being agronomically desirable with large grain and absence of tannins. Thus, sorghum sourced from Ethiopia is a logical choice for breeding improvements.

Early-season cold tolerance in sorghum will not only expand cultivation into temperate regions of the United States, it will also allow the use of stored spring moisture and stabilize sorghum production in fringe areas where severe losses occur due to occasional low temperatures during the growing season. Early-season cold tolerance will also make sorghum an alternative crop in frost-free mild-winter areas, reduce disease and pest pressures^12^, extend the growing season for late cultivars and provide the possibility for a double cropping season.

Low air and soil temperatures adversely affects early sorghum growth and development, and these vary among cultivars for germination, 4.6 to 16.5°C^13,14^, and plumule elongation, 10 to 15.5 °C^13,15^. Field and laboratory tests have revealed genotypic differences for early growth of sorghum under cool conditions^16,17^. The occurrence of low temperatures throughout the growing season have been shown to lengthen growth cycle, delay flowering, induce sterile pollen development, affect pollination, cause reduction of seeds in panicles, and production of sterile pollen during microsporogenesis^18^.

Like most cereal crops, the effect of abiotic stress (temperature, drought, heat etc.) on yield will depend on the intensity, duration, and developmental stage of the crop. Stress, such as drought, at the reproductive stage in sorghum is usually characterized by drastic reduction in grain yields^19,20^. Other stages in sorghum sensitive to abiotic stress includes panicle initiation which occurs during the mid-developmental stage (GSII). Panicle initiation (PI) in sorghum is a very critical phase during vegetative growth, whereby, the sorghum plant transitions from producing leaves to producing a panicle or head. This process also known as growing point differentiation is affected by temperature (assessed by growing degree days; GDD) and photoperiods^17^. Panicle initiation is considered the most critical period of development during which the size of the panicle, the seed number per plant and subsequently the potential yield is set^21^. Seed number per plant accounts for 70 percent of sorghum’s final grain yield. Any factor that affects PI will impede panicle development, thus reducing the number of seed to be formed, and subsequently lowering yields. The maximum potential number of spikelets and seeds per spikelet occurs at this stage where the maximum yield potential is determined over a period of seven to ten days.

Plants require a specific amount of heat to develop from one point of the life cycle to another, such as from seed to panicle initiation in sorghum. This heat unit, otherwise called growing degree unit (GDU), varies with daily temperature. A growing degree day is defined as a day when the average daily temperature is at least one degree above developmental threshold, i.e. the base temperature below which development stops^22^. Growing degree days can be used for crop management especially in fine tuning weed control^21^. Reported the cumulative growing degree units (CGDU) required by grain sorghum at various developmental stages including panicle initiation (942–1365 CGDU) and flowering (1848–1995 CGDU). They showed that this varied with the maturity of the variety.

The effect of temperature and photoperiod on time from planting to PI have been reported in a number of studies both in controlled^23^ and field environments^24,25^. The optimum temperature (T_o_) for PI, as reported by^26^, was assumed to be between 26 and 34 °C. However, data by^23,25^ clearly showed the optimum temperature to be closer to 25–27 °C^17^. Reported T_o_ for PI to be 25 °C, and both cooler and warmer temperatures delayed PI with variability among genotypes. While the effect of suboptimal temperatures on the time to panicle initiation is well known, little to no information is available on how sub-or supra-optimal temperatures affect the panicle size and consequently grain yields in sorghum.

Numerous studies^6,27–33^ screening for early cold tolerance in many plants, including sorghum and maize, have been limited to the seedling stage with final germination percent, seedling emergence percent and index, stand establishment, early vigor ratings, seedling dry weight, chlorophyll content, photosynthetic rate, leaf lipid composition and respiration at low temperatures used as screening tools or characteristics. In the High Plains of West Texas, seedling screening for early cold tolerance in sorghum has been done by early planting in late March or the first week of April, when average daily soil temperatures are usually below 16 °C and fluctuate during the early part of the growing season. The current research aimed to evaluate how early season low temperatures in West Texas affects not only seedling and early plant growth and development characteristics of sorghum, but also the grain yield using a seed-to-seed approach. In addition, the study aimed to characterize the diversity with respect to yield penalties amongst two major sources of cold tolerance in sorghum sown at different planting dates in West Texas.

## Materials and methods

### Plant materials and experimental design

Forty-six grain sorghum lines including hybrids, introgression and recombinant inbred lines (RILs) selected for variability in early season cold tolerance based on field final germination percent, early vigor ratings, and 30-day dry biomass from multi-year field experiments at USDA-ARS, Lubbock, Texas (33.6° N, − 101.9° W), were used in this study. Lines had different sources of seedling cold tolerance including the two major Chinese and Ethiopian background (Table 1). They included: 10 hybrids (denoted CH) from ARS sorghum breeding program developed and selected for seedling cold tolerance, 10 RILs of Chinese-background (denoted HH) from ARS sorghum genetics program, and 14 ARS introgression lines (denoted JB) from the Ethiopian collection. Additionally, 8 commonly used breeding lines, 3 RILs from cross between Chinese cold tolerant Hong-Ki-Ze and BTx623, and 1 commercial hybrid (Pioneer 84P80); *all* with known seedling cold tolerance (CT) and cold susceptibility (CS) were used as checks. Lines were evaluated for the effect of early-planting on yield under variable planting dates using a seed-to-seed approach. Experiments were carried out during the 2017 and 2018 growing seasons on an Amarillo fine sandy loam soil in the research fields at the Cropping Systems Research Laboratory of USDA-ARS in Lubbock, Texas, U.S.A.

**Table 1:** Sorghum lines and hybrids with variable tolerance and susceptibility to early season cold used for characterizing the effects of early, mid, and standard planting dates on productivity in West Texas.

Line ID	Status	Pedigree	Source
JB14	CT	(Combine Kafir 60 x (BTx406 x **PI 452,707**))-6–1-4–4-4–12	Ethiopia
JB15	CT	(Combine Kafir 60 x (BTx406 x **PI 452707**))-6-1-6-1-7-2	Ethiopia
JB17	CT	(RTx436 x (BTx406 x **PI 568,351**))-1-2-13-2-2	Sudan
JB19	CT	(Combine Kafir 60 x (BTx406 x **PI 452,808** ))-2-3-67-2-1-10	Ethiopia
JB20	CT	(Combine Kafir 60 x (BTx406 x **PI 452,808** ))-2-3-81-1-1-1	Ethiopia
JB21	CT	(RTx437 x (BTx406 x **PI 452,605**))-2-4-11-2-3R-2	Ethiopia
JB22	CS	(RTx437 x (BTx406 x **PI 452,605**))-2-4-19-4-2R-2	Ethiopia
JB25	CT	(Combine Kafir 60 x (BTx406 x **PI 452,605**))-1-6 J-2-1-5B-2	Ethiopia
JB26	CT	(Combine Kafir 60 x (BTx406 x **PI 452,605**))-1-6 J-5-4-1B-2	Ethiopia
JB32	CS	(SC414-14E x (BTx406 x **PI 452,808**))-5-10-2-2-1-4	Ethiopia
JB33	CT	(SC414-14E x (BTx406 x **PI 452,808**))-5-10-44-4-3-3	Ethiopia
JB35	CT	(RTx436 x (BTx406 x **PI 452,830**))-1-11-66-4-1-4	Ethiopia
JB43	CT	(RTx437 x (BTx406 x **PI 425,646**))-1-14-1-1-4-1	Ethiopia
JB44	CS	(RTx437 x (BTx406 x **PI 425,646**))-1-14-12-3-3-9	Ethiopia
HH1	CT	(BTx623*PI 568,016)-F2-203-1-1-1-BK	China
HH2	CT	(BTx623*PI 568,016)-F2-193-1-1-1-BK	China
HH3	CT	((p12xp19)-36*BTx642)-F2-44	BTX623 Mutant
HH4	CT	((p12xp19)-110*BTx642)-F2-92	BTX623 Mutant
HH5	CT		China
HH6	CT		China
HH7	CT		China
HH8	CT	BTx623 SCTN	BTX623 Mutant
HH9	CT	20M2-001	BTX623 Mutant
HH10	CT	25M2-1665	BTX623 Mutant
CH1	CT	A.Tx642/R.Tx436	Elite-USA
CH2	CT	A.Tx642/R.Tx436	China
CH3	CT	(A.Tx2752/B.Tx645)/**R.15046**	China
CH4	CT	(A.Tx2752/B.Tx645)/**R.15057**	China
CH5	CT	(A.Tx2752/B.Tx645)/**R.15097**	South America
CH6	CT	(A.Tx2752/B.Tx645)/**R.15112**	Elite-USA
CH7	CT	ADLO357/**R.16006**	Elite-USA
CH8	CT	A3.RTx436/**B.16104**	Elite-USA
CH9	CT	A3.RTx436/**B.16154**	Elite-USA
CH10	CT	(A.Tx2752/B.Tx645)/**R.15097**	Elite-USA
84P80	CT	Commercial hybrid from Pioneer	Pioneer
BTx642	CT	Breeding line	USDA-ARS
BTx623	CS	Breeding line	USDA-ARS
SC1154	CT	Breeding line	USDA-ARS
RTX430	CS	Breeding line	USDA-ARS
RTx2783	CS	Breeding line	USDA-ARS
LBK2	CS	Breeding line	USDA-ARS
LBK1	CS	Breeding line	USDA-ARS
RIL5	CS	**Hong-Ke-Zi** x BTx623	China
RIL42	CT	**Hong-Ke-Zi** x BTx623	China
RIL425	CS	**Hong-Ke-Zi** x BTx623	China
Tx7000	CS	Breeding line	USDA-ARS

Source of cold tolerance in each pedigree is in bold.

*CT* cold tolerant, *CS* cold susceptible.

Sorghum seeds (98,800 seeds/ha) were planted at 3 cm depth, using a modified John Deere MaxEmerge Planter. A randomized complete block design of single-row plots, 5.3 m in length, 1.0 m row spacing and 8–10 cm plant spacing was planted with 60 seeds/plot in 4 replicated plots per line. Pre-plant furrow irrigation plus subsurface drip irrigation of 3 mm/day was applied from planting to physiological maturity.

### Planting dates and growing degree units/days

Based on historical meteorological data, three planting dates: Early (April 1st and March 23rd); average soil temperature usually below 16 °C, Mid (May 1st and May 3rd); average soil temperature at around 20 °C, and Standard (June 1st and June 4th); average soil temperature in mid to upper 20 °C, were used in 2017 and 2018 respectively. Soil temperatures were recorded using data loggers (HOBO Pendant, Onset Computer Corporation, Bourne, MA) randomly placed in the field over the growing season. Air temperatures were recorded on an on-site weather data station at USDA-ARS, Lubbock, Texas. Growth and development stages were predicted using cumulative growing degree units (CGDU) also expressed in growing degree days as described by^21,22^:

$$GDU = \left( {\frac{{T_{max} + T_{min} }}{2}} \right) - T_{base}$$

where GDU is the growing degree unit, T_max_ is the daily temperature maximum, T_min_ is the daily temperature minimum, and T_base_ is the base temperature for sorghum (i.e. 10 °C). Growing degree days were counted as the total number of days in which GDU ≥ 1, and CGDU at a particular developmental stage was the total amount of GDU accumulated up until that given stage.

### Agro-morphological and physiological data collection

Following planting, final germination percent (FGP) was determined at 14 days after planting (DAP) as percent of the number of emerged plumules to the total number of seed planted (60) per plot. Seedling vigor (VGR) was rated visually on a 1–5 scale based on seedling health, stem size, leaf thickness, and length of seedlings of entire plot. A rating of 1 indicated excellent vigor, 2; very good vigor, 3; good vigor, 4; week vigor, and 5; poor vigor. Seedling chlorophyll content (SCC) was measured at 30DAP. Chlorophyll content was also measured at anthesis (ACC). Measurements were taken from the uppermost fully expanded leaf of 5 randomly selected plants per plot using an MC-100 Chlorophyll Concentration Meter (Apogee Instruments, Logan, UT). Shoot of five seedlings were harvested and dried to constant weight to determine seedling dry biomass (SBM).

For mapping of agro-morphological characteristics at physiological maturity, 10–15 randomly selected plants were harvested from the inner 3.6 m of each replicated plot to eliminate edge and saddle-back effects. Characteristics were divided into 2 major categories viz*.* (1) Morphological characters as defined by^19^, such as days to 50% anthesis (DTA), plant height (HGT); from base of plant to tip of panicle, number of leaves (NOL), number of basal tillers (BST), number of nodal (asynchronous) tillers (NDT), stem diameter (SDM); at 30 cm above ground, peduncle length or exertion (PDL); from ear of flag leaf to base of panicle, panicle length (PNL); from base to tip of panicle, panicle width (PWD); measured on the widest section of the panicle, panicle area (PNA); as the product of panicle width and length, AND (2) Yield-related characters as defined by^19^ such as total above ground dry biomass (TBM); weight of chopped plants dried to constant weight at 70 °C, panicle weight (PWT); weight of dried panicle, and panicle harvest index (pHI); the ratio PWT:TBM. Panicle weight was used instead of actual threshed grain yield based on 98% and 91% correlations between panicle weight and threshed grain yields as reported by^19,34^ respectively.

### Statistical analysis

Data were analyzed using SPSS 22.0 (SPSS Inc., USA) and JMP 12 (SAS Institute). Multivariate analysis of variance was used to identify significant interactions of treatments (year, planting dates and genotypes) on assessed characteristics based on Wilk’s Lambda test and its associated significance level expressed in relative partial Eta squared (η^2^). Partial η^2^ represents the proportion of the variance in the dependent variable(s) that can be explained by the independent variable(s). Using^35^, η^2^ ≤ 1% is considered small effect; 1% ≤ η^2^ ≤ 6% is considered medium effect; and η^2^ ≥ 14% is considered a large effect. The Bonferroni adjustment was applied where necessary to avoid type I errors. Tukey’s honest significant difference (HSD) test separated means for assessed parameters. Pearson correlation matrix was used to identify correlations between parameters. Observed trends in ranking of planting dates were based on Tukey–Kramer HSD connecting letters ranking report for the assessed characteristic. Significance was stated at *p* ≤ 0.05 or *p* ≤ 0.01 where applicable. Final germination percent and vigor ratings were transformed using the arcsine^18^ function prior to statistical analysis to minimize the effect of heterogeneity of error variances. The effect of early and mid-planting dates on yield and other assessed parameters was evaluated based on their percent change from standard planting dates.

## Results

### Soil and air temperatures

The optimum temperature for cultivation of sorghum ranges between 25 and 27°/20 °C temperature on a 12/12 photoperiod day/night cycle. While the base soil temperature for sorghum germination is 12 °C, a minimum 15 °C air temperature is required for growth. In the Southern High Plains of West Texas, standard planting date for sorghum is early to mid-June. Field testing for early-season cold tolerance is in late March or the first week of April when average soil temperature is usually at or below 16 °C.

**Figure 1:**
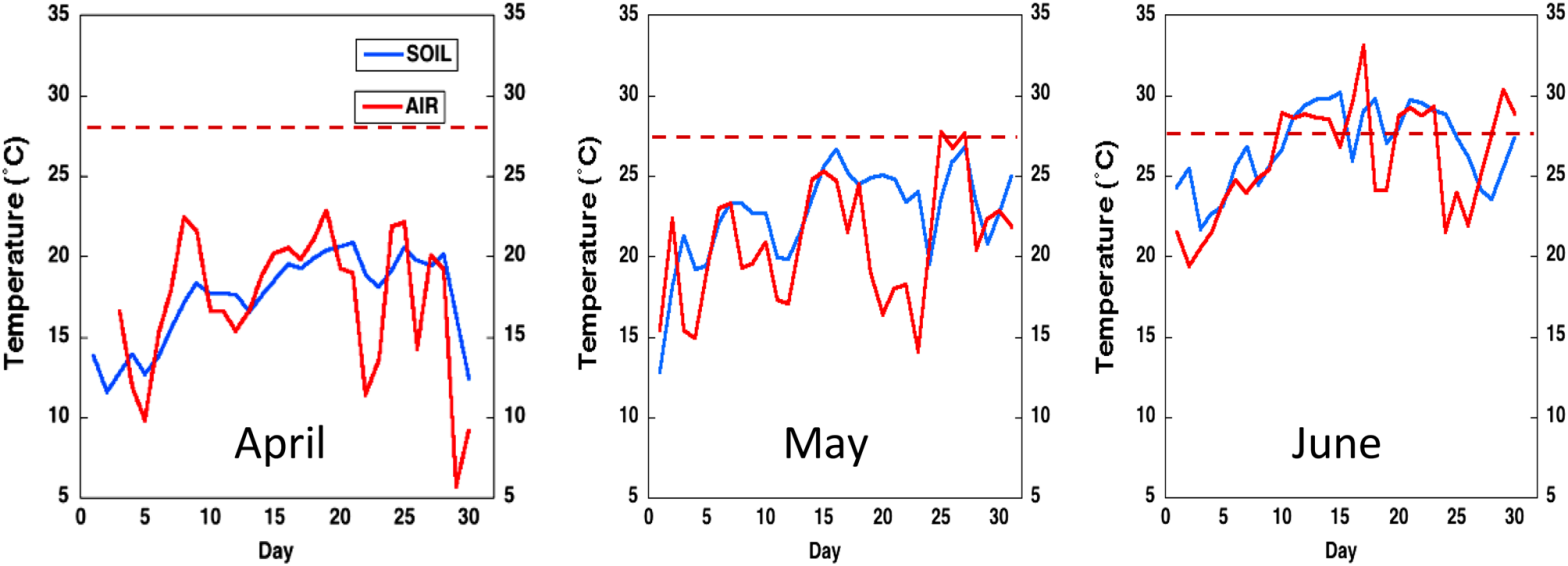
Variations of average early-season soil and air temperatures for three planting dates of grain sorghum in the Southern High Plains of West Texas pooled for 2017 and 2018 seasons. The dotted line represents the optimum temperature for sorghum growth and development.

In the current study (Fig. 1), the average soil temperature for the first 14 days after planting (DAP) in 2017 and 2018 was 14.7 °C. The average day/night air temperature for the was 23/9 °C. Mid-planting was on May 1st with average soil temperature of 20.8 °C in the first 14DAP and a monthly air day/night average of 27/10 °C. Standard planting was on June 1st, with average soil temperature of 27.2 °C and monthly air day/night average of 34/20.

### Data visualization and analysis

To explore strong patterns, identify variations in data set, and explain any variance or covariance in the assessed parameters as a result of variability in years, planting dates or genotypes, multivariate analysis was performed. The effect of independent variables: year, planting dates, and genotypes, showed that year or its interactions with either planting dates or genotypes had mostly small to medium effect on most of the assessed dependent variables (Supplemental Table 1). Thus, data for 2017 and 2018 were pooled for analyses.

Observed variations in agro-morphological parameters (grouped into seedling, plant morphology, and yield related characteristics) based on the treatments (planting dates and genotypes) and their interactive effects were ranked based on partial Eta squared (η^2^) as: small; η^2^ ≤ 1%, medium; 0.01 ≤ η^2^ ≤ 6% and large; η^2^ ≥ 14% (Table 2). Large effects were observed for all assessed parameter across planting dates (except for ACC; η^2^ = 5% and PDL; η^2^ = 0%), genotypes, and their interaction (except for ACC; η^2^ = 13%). Planting date had the strongest effect on seedling parameters with observed η^2^ of 47, 60, 91, and 87% for FGP, VGR, SBM, and SCC respectively. Variations in days to anthesis (DTA) was mostly due to planting dates (η^2^ = 94%) while genotypes affected chlorophyll content at anthesis (ACC; η^2^ = 55%).

**Table 2:** Partial Eta squared (η^2^) values for the individual and interactive effects of planting dates (P) and genotypes (G) on seedling, vegetative, morphology, and yield-related parameters of diverse sorghum lines with variable tolerance to early-season low temperatures, evaluated across three planting dates in West Texas.

	Seedling				Plant morphology											Yield related				
	FGP	VGR	SBM	SCC	ACC	DTA	NOL	HGT	BST	NDT	SDM	PDL	PNL	PNW	PNA	VBM	TBM	PWT	pHI	GRY
P	47^a^	60	91	87	5	94	53	16	63	28	32	0	8	46	37	40	45	23	54	47
G	33	41	30	49	55	70	90	91	61	43	63	26	80	56	63	48	57	57	33	42
P * G	24	16	36	23	13	55	61	32	36	24	29	15	38	31	38	20	36	46	20	34
*Means*	*75*	*2.5*	*3.3*	*377*	*638*	*69*	*16*	*129*	*0.8*	*0.5*	*1.8*	*8.6*	*29*	*5.4*	*158*	*69*	*142*	*73*	*0.39*	*4.5*
*S.E*	*5.01*	*.13*	*.17*	*15.2*	*20.1*	*4.6*	*1.05*	*11.1*	*.02*	*.02*	*.12*	*1.5*	*3.3*	*.47*	*8.7*	*3.4*	*12.2*	*5.2*	*.04*	*.12*

Values are expressed in percentages as proportion of variance in the assessed characteristics explained by the treatments.

*P* planting date, *G* genotype, *FGP* (%) final germination percent, *VGR* vigor rating, *SBM* (g) 30-days old seedling dry biomass, *SCC* (µmolm^−2^) 30-days old seedling chlorophyll content, *ACC* (µmolm^−2^) anthesis chlorophyll content, *DTA* (days) growing degree days to anthesis, *NOL* number of leaves, *HGT* (cm) plant height, *BST* basal tillers, *NDT* nodal tillers, *SDM* (cm) stem diameter, *PDL* (cm) peduncle length, *PNL* (cm) panicle length, *PNW* (cm) panicle width, *PNA* (cm^2^) panicle area, *VBM* (g) vegetative aboveground dry biomass, *TBM* (g) total aboveground dry biomass, *PWT* (g) panicle weight, *pHI* panicle harvest index, *GRY* (ton/ha) grain yield, *SE* standard error.

^a^Indicates using Cohen, 1988, η^2^ ≤ 1% is considered small effect; 0.01 ≤ η^2^ ≤ 6% is considered medium effect; and η^2^ ≥ 14% is considered a large effect.

Variations in morphological parameters (NOL, HGT, BST, NDT, SDM, PDL, PNL, PNW, and PNA) were mostly due to genotypic differences. Observed variations in yield related parameters were highly due to genotypic differences for VBM (η^2^ = 48%), TBM (η^2^ = 57%), PWT (η^2^ = 57%); planting dates for pHI (η^2^ = 54%); and both genotypes (η^2^ = 42%) and planting dates (η^2^ = 47%) for grain yield (GRY).

### Seedling characterization

As in so many current and previous research studies screening for early cold tolerance in sorghum, final germination percent (FGP), early vigor ratings (VGR), 30-DAP seedlings dry weight (SBM) and chlorophyll content (SCC) were analyzed. Based on partial Eta squared analysis (see Table 2)**,** planting dates had the strongest effect on the observed variability in these parameters in the current study (Table 3).

**Table 3:** Seedling characteristics for diverse sorghum lines and hybrids evaluated under three planting dates in West Texas.

Planting dates	FGP (%)	VGR	SBM (g)	SCC (µmol m^−2^)
Early	63.0 B	3.0 A	0.3 C	223 C
Mid	79.0 A	2.6 B	3.8 B	410 B
Standard	82.0 A	1.9 C	5.8 A	497 A
*Mean*	*75.0*	*2.4*	*3.5*	*386*
*SE*	*0.81*	*0.03*	*0.11*	*5.49*

ABC...: indicates significant differences (*p* ≤ 0.05) for assessed parameter across planting dates.

Cooler early temperatures during seed germination and seedling growth significantly (*p* ≤ 0.05) reduced final germination percent (FGP) in early planting (63%) date by 16% and by 19% when compared to mid (79%) and standard (82%) planting dates respectively. 30-day-old seedlings were significantly less vigorous (3.0 vs. 2.6 and 1.9), accumulated less dry biomass (0.3 vs. 3.8 and 5.8 g), and contained less chlorophyll (223 vs. 410 and 497 µmol m^−2^) under early planting date compared to mid and standard planting dates, respectively. All seedling parameters, except FGP, were higher under standard compared to the mid-planting date.

To look at the seedling performance using pre-determined cold status (Table 1), genotypes were pooled into cold tolerant (CT) and cold susceptible (CS) groups. Within and between group comparisons were done for all assessed seedling parameters across planting dates (Table 4). The CT group performed significantly better than the CS group across planting dates with respect to germination (except under standard planting; 83 vs. 82%, ns), vigor, dry biomass, and chlorophyll content (except under mid planting; 412 vs. 408 µmol m^−2^, ns).

**Table 4:** Variations in seedling characteristics within and between cold tolerance (CT) and cold susceptible (CS) groups pooled from a diverse sorghum population planted across three planting dates in West Texas.

Planting dates	FGP (%)		VGR		SBM (g)		SCC (µmol m^−2^)	
	CT	CS	CT	CS	CT	CS	CT	CS
Early	73* B	43 C	2.6* A	3.6 A	0.36* C	0.21 C	230* C	212 C
Mid	84* A	73 B	2.4* A	2.9 B	3.86* B	3.69 B	412^ ns^ B	408 B
Standard	83^ ns^ A	82 A	1.7* B	2.0 C	6.06* A	5.31 A	508* A	482 A
*Mean*	*80*	*68*	*2.3*	*2.8*	*3.5*	*3.4*	*387*	*384*
*SE*	*0.75*	*1.57*	*0.04*	*0.06*	*0.14*	*.15*	*7.31*	*8.30*

*FGP* final germination percent, *VGR* early vigor rating, *SBM* 30DAP dry seedling biomass, *SCC* 30DAP chlorophyll content; ns: non-significant.

*Significant (*p* ≤ 0.05) difference for assessed parameter between groups within planting date.

ABC…: indicates significant difference (*p* ≤ 0.05) for assessed parameter within a group across planting dates.

Both groups generally increased their performance in all assessed seedling parameters as early season temperatures increased from early to mid to standard planting dates. However, no significant difference in germination percent was observed in tolerant genotypes in mid (84%) and standard (83%) planting dates. Likewise, tolerant genotypes showed similar vigor rating for early (2.6) and mid (2.4) planting dates.

### Growing degree days and crop development

Using cumulative growing degree unit (CGDU) ranges for sorghum developmental stages as reported by^21^ (924-1365CGDU for panicle initiation and 1848-1995CGDU for flowering), the current study looked at the effect of planting dates on growing degree days for panicle initiation and flowering (anthesis). The closest CGDUs to the reported range of values was used to determine the range of growing degree days (GDD). The mean GDD (in parentheses) were then statistically compared across planting dates (Table 5).

**Table 5:** Effect of planting dates on crop developmental stages for diverse sorghum lines with variable early-season cold tolerance grown in West Texas.

Developmental stage	Reported range CGDU^a^	Equivalent range of GDD		
		Early planting	Mid planting	Standard planting
Panicle initiation	924–1365	58–74 (66 A)	43–56 (49 B)	30–45 (37 C)
Anthesis	1848–1995	81–83 (82 A)	72–77 (74 B)	60–65 (62 C)

*CGDU* cumulative growing degree units, *GDD* growing degree days.

^a^Values reported by^21^.

ABC…: indicates significant (*p* ≤ 0.05) difference between planting date at particular developmental stage.

On average, early planting significantly delayed panicle initiation by 17 and 29 GDD when compared to mid and standard planting dates, respectively. This resulted in a delay in anthesis on average of 8 and 20 GDD compared to mid and standard planting dates respectively. However, the duration from panicle initiation to anthesis was shorter under early planting (16 GDD) compared to mid (25GDD) and standard (25GDD) planting dates.

To evaluate the effect of planting dates on the development of tolerant (CT) and susceptible (CS) groups, days to 50% visual flowering of lines (excluding hybrids) was calculated. The cumulative growing degree unit at anthesis (CGDU_AN_) was used to determine the amount of growing degree days to anthesis (GDD_AN_), with cumulative growing degree unit and growing degree days at PI (CGDU_PI_ and GDD_PI_ respectively) as baseline (Table 6). Tolerant lines reached flowering significantly earlier under early and mid-planting dates by 6- and 4-degree days, respectively. Days to flowering under standard planting was not significantly different between tolerant and susceptible lines.

**Table 6:** Planting dates effect on growing degree days at 50% flowering of cold tolerant (CT) and susceptible (CS) sorghum lines (excluding hybrids) grown in West Texas.

	Early planting		Mid planting		Standard planting	
	CT	CS	CT	CS	CT	CS
CGDU_PI_	1111	1111	1151	1151	1116	1116
GDD_PI_	66	66	49	49	37	37
CGDU_AN_	1491*	1728	1604*	1741	1780	1843
GDD_AN_	74*	80	65*	69	59	61

*Indicates significance at *p* ≤ 0.05.

### Morphological and Yield related characteristics

Plant morphological traits (except days to anthesis and basal tillers) were generally negatively affected under earlier planting dates (Table 7). Peduncle length averaged 8.5 cm but was not significantly different between planting dates. Early planting dates significantly reduced panicle size (width and area), though effect on panicle length was not significant. Yield related parameters showed a significant trend in reduction from standard to mid to early planting dates. Grain yield from early planting (3.2tons/ha) was 43% and 30% less when compared to standard (5.6tons/ha) and mid-plantings (4.6tons/ha) respectively. Mid-planting yielded 18% less grain than standard planting. Panicle harvest index where significantly higher in early and mid-planting compared to standard planting.

**Table 7:** Morphological and yield related traits of sorghum lines with variable tolerance to early-season cold temperatures evaluated under three planting dates in West Texas.

Category	Parameter	Early	Mid	Standard	Mean ± *S.E*
Plant morphology	Number of leaves	15.2 B	15.6 B	16.6 A	15.9 ± *0.08*
	Days to anthesis (days)	81.7 A	67.5 B	57.7 C	68.2 ± *0.47*
	Plant height (cm)	123.0 B	134.0 A	131.1 A	130.2 ± *1.44*
	Basal tiller	1.7 A	0.6 B	0.2 C	0.8 ± *0.05*
	Nodal tillers	0.2 C	0.5 B	0.9 A	0.6 ± *0.03*
	Stem diameter (cm)	1.6 C	1.8 B	2.0 A	1.9 ± *0.01*
	Peduncle length (cm)	9.5	8.9	7.3	8.5 ± *0.63*
Panicle size	Panicle length (cm)	28.3	29.4	28.8	28.9 ± *0.21*
	Panicle width (cm)	4.3 C	5.4 B	6.1 A	5.4 ± *0.05*
	Panicle area (cm^2^)	135.1 C	162.0 B	177.4 A	159.2 ± *2.13*
Yield related	Panicle weight (g)	65.3 C	72.4 B	83.1 A	74.2 ± *1.18*
	Vegetative biomass (g)	53.4 B	60.1 B	94.3 A	70.2 ± *1.48*
	Grain yield (tons/ha)	3.2 C	4.6 B	5.6 A	4.5 ± 0.09
	Total biomass (g)	118.0 C	132.3 B	177.0 A	144.1 ± *2.20*
	Panicle harvest index	0.56 A	0.54 A	0.48 B	0.39 ± *0.00*

ABC...: indicates significant difference (*p* ≤ 0.05) for assessed parameter within a category across planting dates.

When grouping the lines into tolerant and susceptible groups, most morphological parameters were significantly higher in CT than in CS group under early and mid-plantings dates (Supplemental Table 2). Grain yield was 31% higher in CT (3.6 tons/ha) than CS (2.5 tons/ha) group under early planting date but showed no significant difference (4.6 vs. 4.7 tons/ha) under mid-planting date. Under standard planting dates, only number of leaves and basal tillers were significantly higher for CS than CT group. Grain yield (5.5 vs. 5.7tons/ha) was not significantly different between CT and CS groups, respectively.

### Relationships between assessed agro-morphological characteristics

To evaluate the relationship between assessed parameters, Pearson correlations were performed across planting dates (Supplemental Tables 3, 4, and 5). Assessed parameters were grouped into four main categories: (1) Plant morphological characteristics, (2) Panicle size determining characteristics, (3) Seedling cold tolerance screening characteristics, and (4) Yield related characteristics. The relationships between grain yield and other assessed parameters are outlined in Table 8.

**Table 8:** Pearson correlation between grain yield and other agro-morphological parameters for sorghum lines with variable early-season cold tolerance and susceptibility evaluated at three planting dates in West Texas.

Categories	Assessed parameters	Early planting	Mid planting	Standard Planting
Plant morphology	Number of leaves	ns	ns	0.14*
	Days to flowering	ns	ns	ns
	Plant height	− 0.27**	0.23**	0.15*
	Basal tiller	ns	ns	ns
	Nodal tillers	− 0.13*	− 0.29**	− 0.18*
	Stem diameter	0.14*	0.34**	0.29**
	Peduncle length	− 0.15*	− 0.27**	ns
Panicle size	Panicle length	0.22**	0.39**	0.45**
	Panicle width	0.28**	0.68**	0.76**
	Panicle area	0.29**	0.63**	0.75**
Yield related	Panicle weight	0.51**	0.81**	0.89**
	Vegetative biomass	0.24**	0.45**	0.20**
	Total biomass	0.48**	0.74**	0.63**
	Panicle harvest index	0.28**	0.45**	0.45**
Seedling	Final germ. percent	0.52**	0.29**	0.26**
	Vigor rating	0.41**	0.31**	0.22**
	Seedling chlorophyll	0.16*	ns	ns
	Seedling biomass	0.45**	0.32**	0.29**
	Anthesis chlorophyll	0.22**	ns	ns

**, *Indicate significance at *p* ≤ 0.01 *and p* ≤ 0.05 respectively*.*

Plant morphological parameters showed variable degree of significant relations to grain yield across planting dates. Days to flowering and number of basal tillers were not significantly related to yield. Irrespective of planting dates, number of nodal tillers was negatively related to yield while stem diameter was positively related to yield. Relation between peduncle length and grain yield was negative under early and mid-planting but insignificant under standard planting. Taller plants were negatively related (*r* = − 0.27, *p* ≤ 0.05) to yield under early planting but showed positive relations to yield under mid and standard planting dates.

Panicle size (length, width and area) and yield related parameters showed positive relations to grain yield. The significance of these relationships generally increased from early to mid to standard planting dates. Likewise, seedling parameters such as final germination percent, vigor, and seedling biomass also showed positive relations to yield with the relations being strongest under early planting (*r* = 0.52, 0.41, and 0.45, *p* ≤ 0.05) compared to mid (*r* = 0.29, 0.31, and 0.32, *p* ≤ 0.05) and standard (*r* = 0.26, 0.22, and 0.29, *p* ≤ 0.05) planting dates, respectively. Chlorophyll content for 30-DAP seedling and at anthesis were significantly related to yield only under early planting date.

To further evaluate the estimated contribution (expressed in Beta values) of each of the assessed parameters to the prediction of grain yield, a multiple regression analysis was performed across planting dates with grain yield as the dependent variable to the other predicting variables (Table 9). Together, the assessed parameters predicted grain yield by R-squared values of 94.1%, 97.2%, and 97.7% under early, mid, and standard planting dates respectively. Morphological parameters (except nodal tiller at mid planting) did not provide any significant contribution to predicting grain yield irrespective of planting dates. Panicle length, width, and area were significant traits in predicting grain yield under early and mid- but not under standard planting dates. Final germination percent was a significant predictor of grain yield irrespective of planting dates.

**Table 9:** Standard coefficient estimates (Beta values) from regression results of explanatory agro-morphological parameters on grain yield for sorghum lines with variable early season cold tolerance and susceptibility evaluated at three planting dates in West Texas.

Categories	Assessed parameters	Early planting	Mid planting	Standard Planting
Plant morphology	Number of leaves	0.040	− 0.029	0.005
	Days to flowering	− 0.021	− 0.010	0.003
	Plant height	0.009	− 0.021	− 0.010
	Basal tillers	0.022	− 0.017	− 0.005
	Nodal tillers	− 0.037	− 0.035*	0.013
	Stem diameter	0.029	− 0.011	− 0.015
	Peduncle length	0.035	− 0.001	− 0.006
Panicle size	Panicle length	0.400*	0.497*	0.046
	Panicle width	0.430*	0.552*	0.048
	Panicle area	− 0.697*	− 0.830*	− 0.085
Yield related	Panicle weight	0.850	1.078*	− 0.252
	Vegetative biomass	0.453	0.010	0.622
	Total biomass	− 0.371	− 0.103	0.502
	Panicle harvest index	0.313*	− 0.097	0.122*
Seedling	Final germ. percent	0.907*	0.560*	0.441*
	Vigor rating	0.025	− 0.080	0.001
	Seedling chlorophyll content	0.005	0.030	− 0.007
	Seedling biomass	0.027	− 0.038	0.011
	Anthesis chlorophyll content	0.010	− 0.016	0.000
	*R-squared*	*0.941*	*0.972*	*0.977*

*Indicates parameter’s significant (*p* ≤ 0.05) unique contribution to prediction of grain yield.

### Yield penalty with respect to sources of cold tolerance

Yield penalty was evaluated by comparing reduction in productivity (expressed in percent) per hectare between planting dates. These comparisons were grouped with respect to the different cold tolerant background, to decipher the importance of the origin of cold tolerant source to the breeding for cold tolerance (Table 10). Comparing early versus standard planting dates, yield penalty was lowest for Ethiopian Introgressions (27%) compared to Chinese RILs (37%), BTx642 (41%), commercial cold tolerant Pioneer 84P80 (43%), Chinese hybrids (44%), and susceptible checks (57%). Ethiopian Introgressions also showed the lowest (11%) yield penalty under early versus mid planting dates. Comparing mid versus standard planting dates, yield penalties were statistically similar across Ethiopian introgressions (17%), Chinese RILs (19%) and hybrids (22%), and susceptible checks (20%). BTx642 was indifferent while commercial Pioneer 84P80 showed a 10% yield penalty.

**Table 10:** Yield penalties (expressed in percent grain yield reduction) for sorghum lines with different background source of cold tolerance and susceptibility evaluated at three planting dates in West Texas.

Source of tolerance	Yield penalties (%)		
	Early versus Standard	Early versus Mid	Mid versus Standard
Ethiopian Introgressions	27 C	11 C	17 A
Chinese RILs	37 B	22 B	19 A
BTx642	41 B	40 A	01 C
Chinese Hybrids	44 B	29 B	22 A
Pioneer 84P80	43 B	36 AB	10 B
Susceptible checks	57 A	40 A	20 A

ABC…: indicates significant difference (*p* ≤ 0.05) between sources for a given comparison of planting dates.

## Discussion

Different plant species have minimum, maximum and optimum temperature ranges on which their rate of growth and development depend^36,37^. Sorghum, a tropical cereal known for its drought tolerance, is vulnerable to cooler air and soil temperatures below 15 °C during germination, emergence, and early seedling growth^32,38^. Genetic variability for cold tolerance in sorghum has been detected by early planting under field condition and by seed germination under controlled conditions^8,39,40^. While numerous studies^3,5,16,41^; have recommended germination tests at low temperatures under controlled and field conditions as a screening tool for early establishment in sorghum^42^, reported poor relationship between germination tests in the laboratory and field selection for seedling cold tolerance^43,44^. Concluded that because of its low heritability (or repeatability), germination index has little value for determining sorghum adaptation to a particular temperature.

Variations in genotypic sources of seedling cold tolerance have been reported within the global sorghum germplasm pool. However, the compounded effects of early cold temperatures on vegetative growth and subsequent grain yield have not been characterized. The current study looked at this aspect by evaluating the effects of variable planting dates (early, mid and standard) on sorghum seed germination and establishment, vegetative growth and development and subsequent grain yield of 46 sorghums varieties (including introgressions lines, RILs, hybrids, and cold tolerant and susceptible checks) from diverse sources (including Chinese and Ethiopian) and predetermined degree of seedling tolerance to early season cold temperatures in the Southern High Plains of the United States.

Seedling characteristics such as final germination percent, vigor rating, dry biomass and chlorophyll content at 30-days after planting, showed significant variability across planting dates, with the strongest reductions observed under early planting in April, followed by mid-planting in May and standard planting in June. Generally, cold tolerant lines performed significantly better than susceptible lines. The average soil temperatures during early seedling germination, and the ambient day/night temperatures during establishment and growth, were suboptimal in early (14.7 °C and 23/9 °C) and mid (20.8 °C and 27/10 °C) compared to the optimums in standard (27.2 °C and 34/20 °C) planting dates. Working with 12 sorghum cultivars from the U.S., China, Egypt, South Africa and Rwanda^27^, Reported significant increase in germination, elongation rate and fresh shoot weight with increase in temperature from 5, to 10, 15, and 25 °C. Also^45^, reported that exposure of whole sorghum plant or shoot to a range of 5–15 °C night temperatures, reduced the rate and extent of stomata opening and photosynthesis. The current study showed significant correlation between seedling biomass, vigor and chlorophyll content across planting dates.

Under suboptimal temperatures during the early developmental stages in early and mid-plantings, panicle initiation and flowering were significantly delayed. Panicle initiation (PI) was delayed by 29 and 17 growing degree days (GDD) under early and mid-plantings respectively compared to standard^17^. Reported delay in PI at lower temperatures for nine sorghum genotypes grown in pots in mean temperatures of 17–33 °C, and concluded that the optimum temperature for PI was 25 °C. The duration from PI to flowering was shorter in early (16 GDD) compared to mid (25GDD) and standard (25GDD) plantings. Flowering was delayed by 20 and 8 GDD under early and mid-plantings respectively compared to standard. Thus, following PI, both early and mid-plantings recovered 9 GDD on the standard planting. Tolerant lines flowered 6 and 4 GDD earlier than their susceptible counterparts during early and mid-plantings respectively^46^. Reported that cold tolerant sorghum lines have lower optimum temperatures and higher respiration rates than cold susceptible lines.

While most morphological parameters showed significant relationships to grain yield, their unique contribution to predicting grain yield was insignificant irrespective of planting dates. On the other hand, the size of the panicle (length, width, and area) also showed significant relationships to grain yield but was unique in predicting yield particularly during early and mid-planting dates. That panicle size was able to predict grain yields under sub-optimal temperatures is validated by the findings of^21^ who reported panicle initiation as the most critical period during which the size of the panicle, the seed number per plant and subsequently the potential yield is set. In contrast, of all the seedling parameters measured, only final germination percent had a unique contribution in predicting grain yield across planting dates. This is not surprising as stand counts are an important component of production even under optimal conditions. Additionally, vigor rating, 30-DAP seedling biomass and chlorophyll content, despite their significant relationship to grain yield during early planting, had no unique effect in predicting grain yield across planting dates.

Furthermore, irrespective of sources of cold tolerance, sowing sorghum before the standard planting date in West Texas led to significant yield penalties, effects which were stronger under early than mid planting dates, and also comparatively of lesser magnitude when using Ethiopian sources. Taken together, our results have shown that the deleterious effects of early-season cold temperatures on panicle development persist until maturity and are evident in the grain yield penalties incurred by all germlines that were tested.

## Conclusion

The cultivation of sorghum in high latitudes and temperate regions requires selection and breeding for cold tolerance. Numerous research studies have reported the presence of diversity for seedling cold tolerance in the sorghum germplasm. The reason for cold tolerance differences between Chinese sources and Ethiopian sources of cold tolerance is still not fully understood. Differences in environmental conditions, especially cooler temperatures during anthesis in some regions of Ethiopia may help explain the cold tolerance mechanism differences between China and Ethiopia. Additional studies are needed to fully investigate if different mechanism for cold tolerance exist among these diverse cold tolerant germplasms. The use of characteristics such as final germination percent, vigor rating, chlorophyll content, seedling biomass and other seedlings agro-morphological-and physiological traits, while useful, do not cover the entire developmental spectrum in the selection and breeding for cold tolerance as defined by yield losses. The current study not only showed that early-season cold will result in yield penalty but also reported variability in this trait with respect to the source of cold tolerance. Thus, in developing molecular tools for cold tolerance screening and selection, the compounded impacts of suboptimal thermal growing conditions on grain yield as it relates to critical stages in sorghum growth and development should be taken into consideration.

## Supplementary Information

Supplementary Information
