# Supplementary material for: Seed-to-seed early-season cold resiliency in sorghum
Source: Sci Rep. 2021 Apr 8;11:7801. doi: 10.1038/s41598-021-87450-1 (PMC8032771; doi:10.1038/s41598-021-87450-1)
Supplement: Supplementary file 1 — Supplementary Information [file 41598_2021_87450_MOESM1_ESM.docx]

Supplementary Information

Manuscript title: Seed-to-seed Early-season Cold Resiliency in Sorghum.

Yves Emendack^1*^, Jacobo Sanchez^1^, Chad Hayes^1^, Matthew Nesbitt^1^, Haydee Laza^2^, John Burke^3^

^1^USDA-ARS, Cropping Systems Research Laboratory, Lubbock, TX, USA.

^2^Texas Tech University, Department of Plant and Soil Sciences, Lubbock, TX, USA.

^3^USDA-ARS, Cropping Systems Research Laboratory, Lubbock, TX, USA. (Retired)

*Corresponding author, E-mail: [Yves.Emendack@ars.usda.gov](mailto:Yves.Emendack@ars.usda.gov); 3810 4^th^ Street Lubbock, Texas, United States. Telephone: +1 (806) 723-5224.

Supplementary Table 1: Partial Eta squared (η^2^) values for the individual and interactive effects of year (Y), planting dates (P) and genotypes (G) on seedling, vegetative, morphological, and yield-related parameters of diverse sorghum lines with variable tolerance to early-season low temperatures evaluated across three planting dates in West Texas. Values are expressed in percentages as proportion of variance in the assessed characteristics explained by the treatments.

|  | Seedling | | | | Plant morphology | | | | | | | | | | | Yield related | | | | |
| --- | --- | --- | --- | --- | --- | --- | --- | --- | --- | --- | --- | --- | --- | --- | --- | --- | --- | --- | --- | --- |
|  | FGP | VGR | SBM | SCC | ACC | DTA | NOL | HGT | BST | NDT | SDM | PDL | PNL | PNW | PNA | VBM | TBM | PWT | pHI | GRY |
| Y | ^Ψ^4 | 1 | 3 | 22 | 14 | 12 | 1 | 5 | 11 | 8 | 2 | 0 | 1 | 6 | 6 | 12 | 7 | 8 | 10 | 6 |
| P | 38 | 55 | 92 | 87 | 7 | 94 | 51 | 13 | 60 | 25 | 32 | 1 | 4 | 42 | 32 | 41 | 41 | 21 | 55 | 43 |
| G | 44 | 58 | 48 | 51 | 55 | 70 | 89 | 91 | 59 | 53 | 64 | 28 | 79 | 64 | 75 | 49 | 54 | 68 | 60 | 58 |
| P * G | 40 | 23 | 52 | 26 | 15 | 57 | 59 | 34 | 41 | 28 | 31 | 17 | 32 | 28 | 38 | 21 | 31 | 41 | 31 | 38 |
| *Means* | *68* | *2.2* | *3.9* | *402* | *661* | *64* | *16* | *132* | *0.5* | *0.3* | *2.1* | *7.8* | *28* | *5.1* | *145* | *61* | *122* | *67* | *0.37* | *4.7* |
| *S.E.* | *7.21* | *.20* | *.27* | *13.6* | *40.7* | *3.2* | *0.85* | *9.7* | *.04* | *.00* | *.09* | *3.1* | *3.6* | *.29* | *10.7* | *13.0* | *17.7* | *7.0* | *.02* | *.08* |

Y: year; P: planting date; G: genotype; FGP (%): final germination percent; VGR: vigor rating; SBM (g): 30-days old seedling dry biomass; SCL (µmolm^-2^): 30-days old seedling chlorophyll content; ACC (µmolm^-2^): anthesis chlorophyll content; DTA (days): growing degree days to anthesis; NOL: number of leaves; HGT (cm): plant height; BST: basal tillers; NDT: nodal tillers; SDM (cm): stem diameter; PDL (cm): peduncle length; PNL (cm): panicle length; PNW (cm): panicle width; PNA (cm^2^): panicle area; VBM (g): vegetative aboveground dry biomass; TBM (g): total aboveground dry biomass; PWT (g): panicle weight; pHI: panicle harvest index; GRY (ton/ha): grain yield; *SE: standard error*. ^Ψ^ indicates using Cohen, 1988, η^2^≤1% is considered small effect; 0.01≤η^2^≤6% is considered medium effect; and η^2^≥14% is considered a large effect.

Supplemental Table 2: Morphological and yield related traits of sorghum lines with variable tolerance to seedling cold temperatures evaluated under three planting dates in West Texas.

| Planting  dates | Status | Plant morphology | | | | | | | | | | Yield related | | | | |
| --- | --- | --- | --- | --- | --- | --- | --- | --- | --- | --- | --- | --- | --- | --- | --- | --- |
|  |  | DTA | NOL | HGT | BST | NDT | SDM | PDL | PNL | PNW | PNA | VBM | TBM | PWT | pHI | GRY |
| Early | CT | 80* | 15.0* | 129* | 1.6* | 0.3* | 1.7* | 10.3* | 27.6* | 5.0* | 148* | 54 | 124 | 73* | 0.59* | 3.6* |
|  | CS | 84 | 15.6 | 111 | 2.0 | 0.0 | 1.8 | 8.0 | 29.0 | 4.1 | 128 | 52 | 115 | 60 | 0.52 | 2.5 |
| Mid | CT | 67* | 15.5 | 136 | 0.4* | 0.7* | 1.8* | 10.1* | 28.4* | 5.8* | 181* | 59 | 145* | 79* | 0.55 | 4.6 |
|  | CS | 69 | 15.8 | 131 | 0.8 | 0.3 | 1.9 | 7.3 | 30.9 | 5.1 | 149 | 64 | 126 | 68 | 0.54 | 4.7 |
| Standard | CT | 57 | 16.3* | 134 | 0.1* | 1.0 | 2.0 | 6.3 | 28.8 | 6.0 | 176 | 93 | 174 | 81 | 0.47 | 5.5 |
|  | CS | 58 | 17.1 | 127 | 0.3 | 0.8 | 2.0 | 7.6 | 29.2 | 6.3 | 182 | 96 | 181 | 84 | 0.48 | 5.7 |

*indicates value is significantly different (*p≤0.05*) between cold tolerant (CT) and cold susceptible (CS) groups for a given parameter under a specific planting date. DTA: days to anthesis; NOL: number of leaves; HGT (cm): plant height; BST: basal tillers; NDT: nodal tillers; SDM (cm): stem diameter; PDL (cm): peduncle length; PNL (cm): panicle length; PNW (cm): panicle width; PNA (cm^2^): panicle area; VBM (g): vegetative aboveground dry biomass; TBM (g): total aboveground dry biomass; PWT (g): panicle weight; pHI: panicle harvest index; GRY (ton/ha): grain yield.

Supplemental Table 3: Pearson correlation matrix for agro-morphological and physiological parameters of sorghum lines with variable early season cold tolerance and susceptibility subjected to early planting in West Texas.

| Early | NOL | DTF | HGT | BST | NDT | SDN | PDL | PNL | PNW | PNA | VBM | PWT | GRY | TBM | pHI | FGP | VGR | SCC | ACC | SBM |
| --- | --- | --- | --- | --- | --- | --- | --- | --- | --- | --- | --- | --- | --- | --- | --- | --- | --- | --- | --- | --- |
| NOL |  |  |  |  |  |  |  |  |  |  |  |  |  |  |  |  |  |  |  |  |
| DTF | 0.67** |  |  |  |  |  |  |  |  |  |  |  |  |  |  |  |  |  |  |  |
| HGT |  | 0.15* |  |  |  |  |  |  |  |  |  |  |  |  |  |  |  |  |  |  |
| BST |  |  | -0.28** |  |  |  |  |  |  |  |  |  |  |  |  |  |  |  |  |  |
| NDT | -0.28** | -0.14* | 0.38** |  |  |  |  |  |  |  |  |  |  |  |  |  |  |  |  |  |
| SDM | 0.61** | 0.47** | -0.18* | -0.14* | -0.33** |  |  |  |  |  |  |  |  |  |  |  |  |  |  |  |
| PDL | -0.32** | -0.19* |  | -0.15* | 0.19* | -0.24** |  |  |  |  |  |  |  |  |  |  |  |  |  |  |
| PNL | 0.28** |  |  |  | -0.41** | 0.33** | -0.15** |  |  |  |  |  |  |  |  |  |  |  |  |  |
| PNW | 0.19* |  |  |  | -0.24** | 0.29** | -0.42** | 0.43** |  |  |  |  |  |  |  |  |  |  |  |  |
| PNA | 0.27** |  |  |  | -0.37** | 0.35** | -0.35** | 0.82** | 0.86** |  |  |  |  |  |  |  |  |  |  |  |
| VBM | 0.41** | 0.43** | 0.47** | -0.16* |  | 0.35** |  |  | 0.18* | 0.17* |  |  |  |  |  |  |  |  |  |  |
| PWT |  |  |  |  | -0.36** | 0.24** | -0.39** | 0.39** | 0.66** | 0.63** | 0.32** |  |  |  |  |  |  |  |  |  |
| GRY |  | -0.27 |  |  | -0.13* | 0.14** | -0.15* | 0.21** | 0.28** | 0.29** | 0.24** | 0.51** |  |  |  |  |  |  |  |  |
| TBM | 0.31** | 0.21 | 0.24** |  | -0.27** | 0.35** | -0.30** | 0.31** | 0.55** | 0.51** | 0.78** | 0.85 | 0.48** |  |  |  |  |  |  |  |
| pHI | -0.18* | -0.34 | -0.43** | 0.16* | -0.36** |  | -0.34** | 0.32** | 0.46** | 0.45 | -0.51 | 0.61 | 0.28** |  |  |  |  |  |  |  |
| FGP | -0.19* | -0.28 | 0.13* | -0.18* | 0.29** | -0.14* | 0.23** | -0.20** | -0.36** | -0.32 |  | -0.42 | 0.52** | -0.31** | -0.33** |  |  |  |  |  |
| VGR |  | 0.28 | -0.29** |  | -0.22** |  | -0.22** |  | 0.21** | 0.17 | -0.19 | 0.26 | 0.41** |  | 0.39** | -0.71** |  |  |  |  |
| SCC |  |  |  | 0.14* | 0.12* |  | 0.23** |  | -0.13* | -0.12 |  |  | 0.16* |  |  | 0.27** | 0.31** |  |  |  |
| ACC | -0.44** | -0.67** | -0.30** | 0.15* | 0.19* | -0.33** | 0.15* |  |  |  | -0.47 |  | 0.22** | -0.25** | 0.37** | 0.24** |  | 0.37** |  |  |
| SBM |  | -0.27** |  |  |  |  |  |  |  |  |  |  | 0.45** |  |  | 0.47** | 0.64** | 0.36** | 0.30** |  |

Supplemental Table 4: Pearson correlation matrix for agro-morphological and physiological parameters of sorghum lines with variable early season cold tolerance and susceptibility subjected to mid planting in West Texas.

| Mid | NOL | DTF | HGT | BST | NDT | SDN | PDL | PNL | PNW | PNA | VBM | PWT | GRY | TBM | pHI | FGP | VGR | SCC | ACC | SBM |
| --- | --- | --- | --- | --- | --- | --- | --- | --- | --- | --- | --- | --- | --- | --- | --- | --- | --- | --- | --- | --- |
| NOL |  |  |  |  |  |  |  |  |  |  |  |  |  |  |  |  |  |  |  |  |
| DTF | 0.77** |  |  |  |  |  |  |  |  |  |  |  |  |  |  |  |  |  |  |  |
| HGT |  |  |  |  |  |  |  |  |  |  |  |  |  |  |  |  |  |  |  |  |
| BST |  |  |  |  |  |  |  |  |  |  |  |  |  |  |  |  |  |  |  |  |
| NDT | -0.35** | -0.45** |  |  |  |  |  |  |  |  |  |  |  |  |  |  |  |  |  |  |
| SDM | 0.52** | 0.45** | -0.17* |  | -0.33** |  |  |  |  |  |  |  |  |  |  |  |  |  |  |  |
| PDL | -0.29 | -0.32** |  |  | 0.33** | -0.27** |  |  |  |  |  |  |  |  |  |  |  |  |  |  |
| PNL |  |  |  |  | -0.40** | 0.42** | -0.24** |  |  |  |  |  |  |  |  |  |  |  |  |  |
| PNW | 0.23** | 0.24** | 0.32** |  | -0.37** | 0.38** | -0.36** | 0.40** |  |  |  |  |  |  |  |  |  |  |  |  |
| PNA | 0.15* | 0.17* | 0.19* |  | -0.43** | 0.46** | -0.35** | 0.81** | 0.85** |  |  |  |  |  |  |  |  |  |  |  |
| VBM | 0.45** | 0.47** | 0.68** |  | -0.30** | 0.44** | -0.27** | 0.17** | 0.55** | 0.45** |  |  |  |  |  |  |  |  |  |  |
| PWT | 0.15* |  | 0.20** |  | -0.40** | 0.46** | -0.36** | 0.51** | 0.84** | 0.81** | 0.51** |  |  |  |  |  |  |  |  |  |
| GRY |  |  | 0.23** |  | -0.29** | 0.34** | -0.27** | 0.39** | 0.68** | 0.63** | 0.45** | 0.81** |  |  |  |  |  |  |  |  |
| TBM | 0.33** | 0.32** | 0.49** |  | -0.41** | 0.52** | -0.37** | 0.42** | 0.81** | 0.74** | 0.84** | 0.89** | 0.74** |  |  |  |  |  |  |  |
| pHI | -0.19* | -0.26** | -0.39** | 0.17* | -0.23** |  | -0.20** | 0.41** | 0.43** | 0.46** | -0.34** | 0.58** | 0.45** | 0.18* |  |  |  |  |  |  |
| FGP | -0.16* | -0.21* |  | -0.38** | 0.26** | -0.23** | 0.18* | -0.23** | -0.27** | -0.29** |  | -0.30** | 0.29** | -0.25** | -0.19* |  |  |  |  |  |
| VGR |  | -0.27** |  | -0.21** | 0.26** |  |  | -0.16* |  |  |  |  | 0.31** |  |  | 0.51** |  |  |  |  |
| SCC |  | -0.16* | -0.13* | 0.29** |  |  | 0.24** |  |  |  |  |  |  |  |  |  | 0.24** |  |  |  |
| ACC | -0.51** | -0.62** | -0.22** | 0.26** | 0.23** | -0.25** | 0.40** |  | -0.22** | -0.16* | -0.43** |  |  | -0.28** | 0.28** |  | 0.15* | 0.45** |  |  |
| SBM |  | -0.26** |  |  | 0.14* |  |  |  |  |  |  | 0.17* | 0.32 |  | 0.12* | 0.25** | 0.68** | 0.47** | 0.24** |  |

Supplemental Table 5: Pearson correlation matrix for agro-morphological and physiological parameters of sorghum lines with variable early season cold tolerance and susceptibility subjected to standard planting in West Texas.

| Stand. | NOL | DTF | HGT | BST | NDT | SDN | PDL | PNL | PNW | PNA | VBM | PWT | GRY | TBM | pHI | FGP | VGR | SCC | ACC | SBM |
| --- | --- | --- | --- | --- | --- | --- | --- | --- | --- | --- | --- | --- | --- | --- | --- | --- | --- | --- | --- | --- |
| NOL |  |  |  |  |  |  |  |  |  |  |  |  |  |  |  |  |  |  |  |  |
| DTF | 0.23** |  |  |  |  |  |  |  |  |  |  |  |  |  |  |  |  |  |  |  |
| HGT |  | 0.19* |  |  |  |  |  |  |  |  |  |  |  |  |  |  |  |  |  |  |
| BST | -0.13* |  | -0.16* |  |  |  |  |  |  |  |  |  |  |  |  |  |  |  |  |  |
| NDT | -0.13* |  |  | -0.15* |  |  |  |  |  |  |  |  |  |  |  |  |  |  |  |  |
| SDM | 0.34** |  | -0.16* | -0.19* |  |  |  |  |  |  |  |  |  |  |  |  |  |  |  |  |
| PDL |  |  |  |  |  |  |  |  |  |  |  |  |  |  |  |  |  |  |  |  |
| PNL |  | -0.16* |  |  | -0.13* | 0.41** |  |  |  |  |  |  |  |  |  |  |  |  |  |  |
| PNW | 0.17* |  | 0.37** |  | -0.27** | 0.19** |  | 0.28** |  |  |  |  |  |  |  |  |  |  |  |  |
| PNA |  |  | 0.16* |  | -0.24** | 0.38** |  | 0.80** | 0.79** |  |  |  |  |  |  |  |  |  |  |  |
| VBM | 0.34** | 0.16* | 0.44** | -0.15* |  | 0.30** |  |  | 0.26** |  |  |  |  |  |  |  |  |  |  |  |
| PWT | 0.15* |  | 0.18* |  | -0.26** | 0.32** |  | 0.50** | 0.86** | 0.84 | 0.16* |  |  |  |  |  |  |  |  |  |
| GRY | 0.14* |  | 0.15* |  | -0.18* | 0.29** |  | 0.45** | 0.76** | 0.75 | 0.20** | 0.89** |  |  |  |  |  |  |  |  |
| TBM | 0.34** | 0.13* | 0.43** |  | -0.17* | 0.40** |  | 0.26** | 0.67** | 0.57 | 0.85** | 0.66** | 0.63** |  |  |  |  |  |  |  |
| pHI |  |  | -0.18* | 0.15* | -0.22** |  |  | 0.36** | 0.41** | 0.47 | -0.69** | 0.56** | 0.45** | -0.22** |  |  |  |  |  |  |
| FGP |  |  |  |  | 0.16* |  |  |  | -0.17* | -0.14 |  | -0.19* | 0.26** |  | -0.18* |  |  |  |  |  |
| VGR |  |  |  | -0.23** | 0.14* | 0.24** |  |  | 0.14* | 0.14 |  | 0.21** | 0.22** | 0.15* |  |  |  |  |  |  |
| SCC | -0.20* |  |  |  |  |  |  | 0.15* |  |  |  |  |  |  |  |  | 0.21** |  |  |  |
| ACC | -0.46* | -0.23** | -0.33** | 0.20** |  |  |  |  | -0.18* |  | -0.38** |  |  | -0.32** | 0.21** |  |  | 0.45** |  |  |
| SBM |  |  | 0.13* |  |  | 0.21** |  |  | 0.24** | 0.21** |  | 0.29** | 0.29** | 0.21** |  |  | 0.43** | 0.31** | 0.14** |  |
